# Health claims on foods: challenge for clinical research companies

**DOI:** 10.3402/mehd.v23i0.18739

**Published:** 2012-06-18

**Authors:** Essi Sarkkinen, Pia Karjalainen, Mari Lyyra

**Affiliations:** Oy Foodfiles Ltd, Neulaniementie, Kuopio, Finland

## Abstract

**Background:**

The Nutrition and Health Claim Regulation 1924/2006/EC, together with EFSA guidances on the scientific requirements for different type of health claims, is setting the basis for health claim substantiation in the EU.

**Aim:**

The aim of this presentation is to bring up the key challenges that the food industry and clinical research organizations are facing when meeting these requirements.

**Results and discussion:**

Key issues in clinical research planning to meet the requirements set for the health claim substantiation are: (1) Selection of right outcome markers since the selection of outcome marker defines actually the formulation of the health claim to be used on food or food ingredient. (2) Selection of right target population since that determines the target consumer group for the food with a health claim. (3) Selection of dose regime and food matrices used since these largely determine the conditions set for the use of the health claim. One of the major challenges in health claim substantiation is the deviant approach to risk factors or biomarkers. From the regulation point of view, a single risk factor approach is emphasized, but from the clinical and scientific point of view the pattern of different risk markers or biomarkers could, in some cases, be a more relevant choice to reflect the final health outcome. This is especially the case in the nutrition and health area because we are often dealing with weak but multiple health effects of certain food items or ingredients. Also the lack of validated well-established biomarkers potent to be affected by diet is a challenge in health claim substantiation.

The selection of right target population is often a compromise between choosing a more potential target group to obtain efficacy (i.e. risk factors elevated vs. patient groups) and choosing a rationale to generalize the results to wider population (target consumer) group.

The selection of optimal dosing regime and matrices for a clinical study is partly dependent on previous scientific data on the dose response, if existing. But equally important is the choice of feasible doses from product formulation and food consumption point of view.

**Conclusion:**

With careful analysis of the existing data, advance planning and clinical research strategy it is possible to build up a health claim substantiation that meets the requirements both of Nutrition and Health Claim Regulation and EFSA.
